# Supplementary material for: Scribble Controls Social Motivation Behavior through the Regulation of the ERK/Mnk1 Pathway
Source: Cells. 2022 May 10;11(10):1601. doi: 10.3390/cells11101601 (PMC9139383; doi:10.3390/cells11101601)
Supplement: Supplementary file 1 [file cells-11-01601-s001.zip › cells-1695184-supplementary.pdf]

Scribble controls social motivation behavior through the regulation of the ERK/Mnk1 pathway

Maïté M. Moreau[1], Susanna Pietropaolo[2], Jérôme Ezan[1], Benjamin J.A. Robert[1], Sylvain Miraux[3], Marlène Maître[1], Yoon Cho[2], Wim E. Crusio[2], Mireille Montcouquiol[1*] & Nathalie Sans[1*]

1. Univ. Bordeaux, INSERM, Neurocentre Magendie, U1215, F-33077 Bordeaux, France
2. Univ. Bordeaux, CNRS, Aquitaine Institute for Cognitive and Integrative Neurosciences, UMR5287, F-33405 Bordeaux, France
3. Univ. Bordeaux, Centre de Résonance Magnétique des Systèmes Biologiques UMR5536, F-33077 Bordeaux, France

Correspondence : Dr N. Sans or Dr. M.M. Moreau, Neurocentre Magendie U1215, Planar Polarity and Plasticity team, 146 rue Léo-Saignat, 33077 Bordeaux Cedex, France. E-mail: nathalie.sans@inserm.fr & maite.moreau@inserm.fr

* These senior authors contributed equally to this work

Supplementary Information
Supplementary Tables

■ Supplementary Table S1. *Scrib1*$^{crc/+}$ mice have normal basic motor functions, exploratory behavior, and anxiety

The *Scrib*$^{crc/+}$ mice showed normal rearing and self-grooming in their home cages, similar to control mice. Also, no differences between groups were found when comparing the number of repetitive entries in the same arm of the Y-maze test, and the number of repetitive digging behavior in the marble-burying test.

	WT	*Scrib1*$^{crc/+}$	*t*-test
Physical characteristics			
Body weight (gr)	29.10 ± 0.55	28.50 ± 0.76	t_{30} = 0.61, n.s
Locomotor activity			
Open field : Total distance (cm/30min)	9401.6 ± 434.4	10519.6 ± 6	t_{22} = 1.19, n.s
Plus maze : Total distance (cm/5min)	807.2 ± 61.9	810.0 ± 50.3	t_{23} = 0.03, n.s
Repetitive behavior			
Spontaneous alternation Y maze (%)	69.40 ± 2.55	70.60 ± 1.83	t_{23} = 0.37, n.s
Self-grooming (sec/10 min)	63.87 ± 15.13	51.70 ± 10.03	t_{23} = 0.68, n.s
Rearing (sec/10 min)	64.49 ± 8.676	64.34 ± 9.74	t_{23} = 0.011, n.s
Marble buried (number/30min)	13.56 ± 1.88	14.70 ± 1.726	t_{23} = 0.44, n.s
Neophobia			
Time around the object (sec/30min)	527.80 ± 113.3	429.80 ± 67.4	t_{22} = 0.78, n.s
Distance around the object (cm/30min)	2529.5 ± 354.4	2058.5 ± 259.9	t_{22} = 1.05, n.s
Number of entries around the object	67.25 ± 8.85	66.00 ± 5.57	t_{22} = 0.12, n.s
Anxiety			
Open field : Time in center (sec/30min)	146.8 ± 11.5	148.8 ± 18.4	t_{22} = 0.07, n.s
Open field : Distance in center (cm/30min)	1638.1 ± 152.1	1809.9 ± 227.2	t_{22} = 0.50, n.s
Plus maze : % Time in open arm (5min)	15.69 ± 3.57	13.72 ± 2.70	t_{23} = 0.43, n.s
Plus maze : Number of entries (5min)	15.77 ± 1.74	18.08 ± 1.81	t_{23} = 0.91, n.s
Light/Dark : latency to emerge (15min)	112.13 ± 29.73	112.19 ± 56.01	t_{22} = 0.12, n.s
Light/Dark : Time inside (15min)	342.25 ± 64.28	346.56 ± 54.37	t_{22} = 0.04, n.s
Light/Dark : Number of entries (15min)	14.37 ± 1.73	16.62 ± 1.87	t_{22} = 0.76, n.s

All data are presented as Mean ± SEM. No significant effect of genotype was observed (all n.s.)

■ **Supplementary Table S2.** ***Scrib1**$^{crc/+}$* **mice have normal pre pulse inhibition**

Using the startle response and prepulse inhibition (PPI), we found that *Scrib*$^{crc/+}$ had normal sensory-motor gating (pulse effect for WT and *Scrib*$^{crc/+}$respectively, $F_{2,4}$ = 269.98 and 10.05, n.s).

Arbitrary units for startle; %PPI for the rest:

		lnp100	lnp110	lnp120	perc_PPI_100	perc_PPI_110	perc_PPI_120
**WT**	mean	2.43	3.42	4.54	13.41	33.24	26.95
	SEM	*0.*07	*0.*10	*0.*12	5.61	6.04	5.90
***Scrib1**$^{crc/+}$*	mean	2.50	3.76	4.88	8.99	41.61	29.91
	SEM	*0.*12	*0.*16	*0.*15	7.49	4.82	5.70

■ **Supplementary Table S3. Measure of change in Zif268 immunoreactive cells in response to social test (cells/mm² x10)**

	**WT**	***Scrib1**$^{crc/+}$*	***p***
**Unexposed**			
**DG**	5.91 ± 1.45	4.97 ± 1.33	*n.s*
**CA1**	15.36 ± 3.77	17.59 ± 59	*n.s*
**CA3**	2.38 ± 1.10	3.65 ± 2.09	*n.s*
**Social test**			
**DG**	24.05 ± 2.62	39.54 ± 6.17	$p < 0.05$
**CA1**	36.01 ± 3.48	36.74 ± 3.31	*n.s*
**CA3**	12.85 ± 1.25	22.65 ± 3.83	$p < 0.05$

All data are presented as Mean ±SEM. * $p \leq 0.05$. No significant effect of genotype was observed (n.s.)

Supplementary Figures

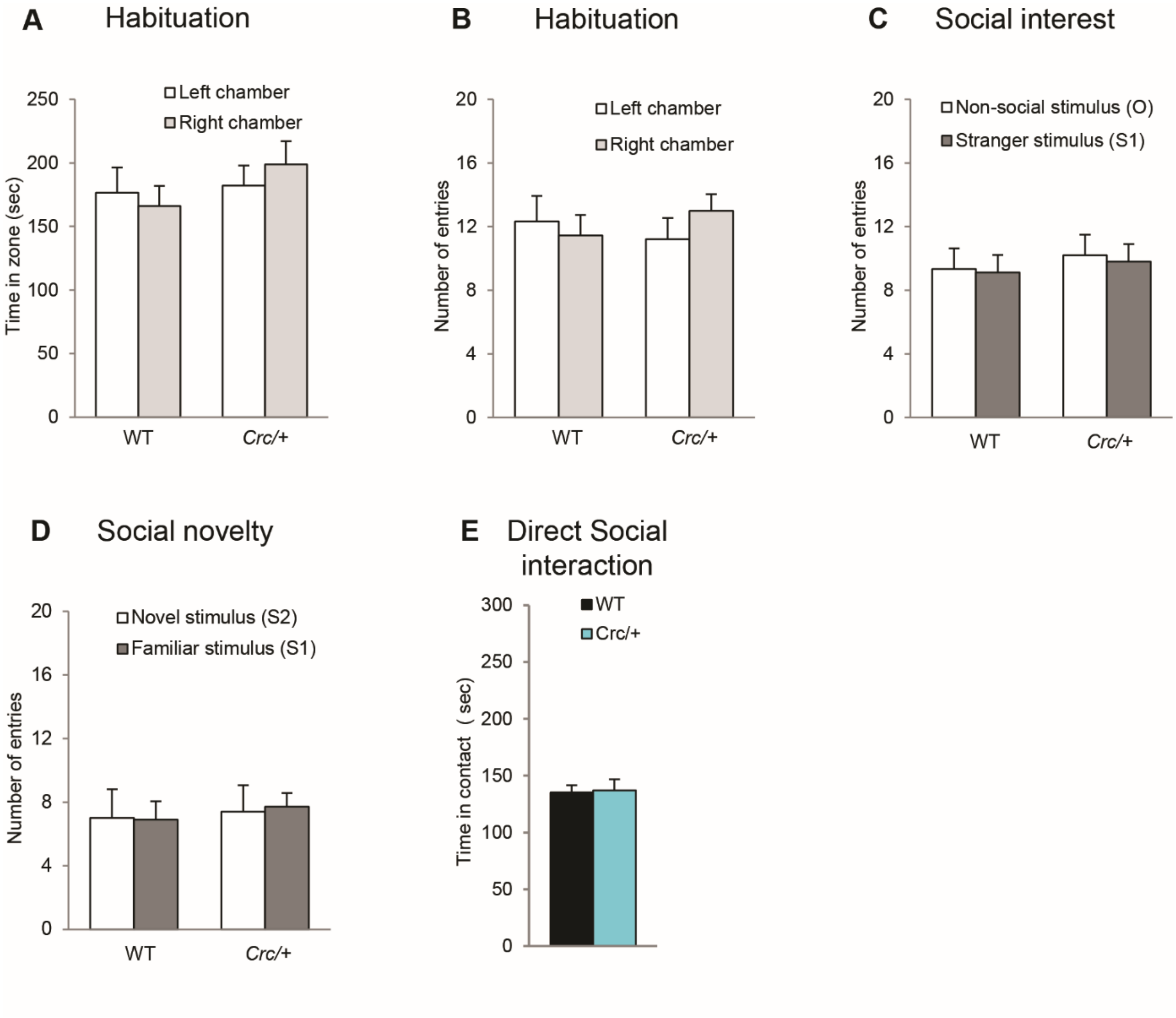

Supplementary Figure S1. The social behavior deficit of the *Scrib1*$^{crc/+}$ mice is not due to an activity deficit. (**A**) WT and *Scrib1*$^{crc/+}$ mice spend the same time (no genotype x chamber interaction: $F_{1,34} < 1$, n.s) and (**B**) perform the same number of entries in the different chamber during the habituation test (n= 9-10 animals per genotype). (**C**) WT and *Scrib1*$^{crc/+}$ mice perform the same number of entries in social stimulus chamber (S1) versus empty chamber (non-social stimulus, O) during the social interest and (**D**) the Social novelty test (n= 9-10 animals per genotype). (**E**) WT and *Scrib1*$^{crc/+}$ mice perform the same number of contact direct social interaction test (t_{24} = 0.16, n.s ; n= 12-14 animals per genotype)

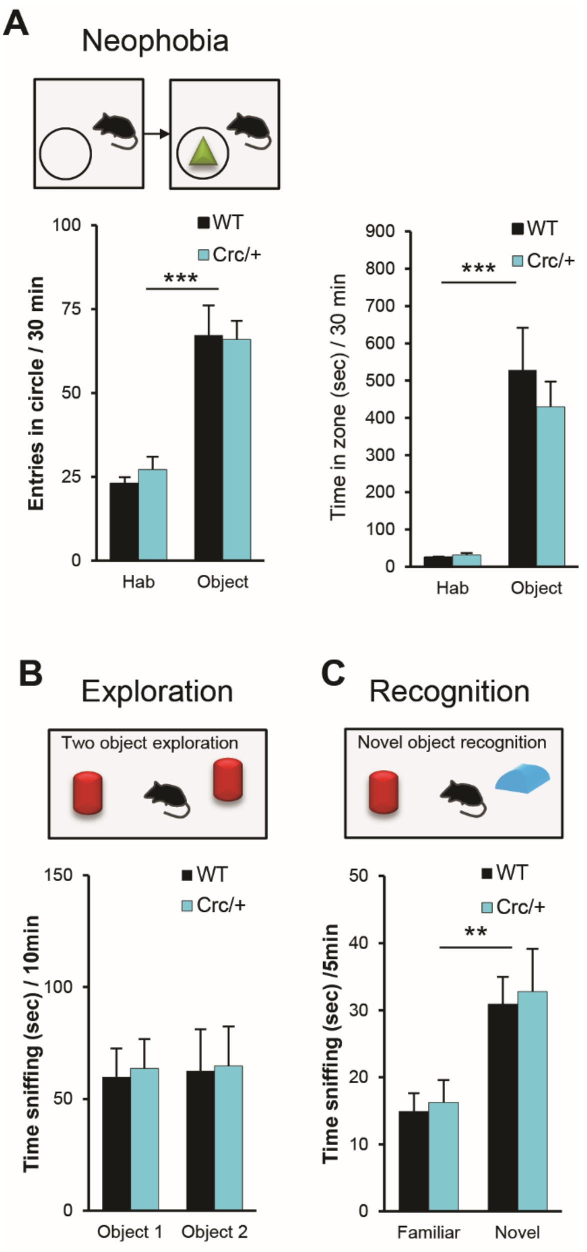

Supplementary Figure S2. The social behavior deficit of the *Scrib1$^{crc/+}$* mice is not due to abnormal general novelty recognition. (**A**) Experimental design protocol of the open field and neophobia test. We used a familiar open field environment to ascertain the intact abilities of WT and mutant mice to explore novel objects. Number of entries in the area which contain the object during the simple open field exploration (habituation) and the neophobia test (Object). In the open field, both WT and *Scrib$^{crc/+}$* mice completed a high number of entries and spent more time in the center after introduction of the object (effect of object: $F_{1,44}$ = 52.23, *** P < 0.0001 for entries; $F_{1,44}$ = 46.96, *** P < 0.0001 for time; no genotype effect: $F_{1,44}$ = .75, n.s for time; $F_{1,44}$ = 0.06, n.s for entries; n=8-16 animals per genotype), confirming that the novel object elicited curiosity. (**B,C**) Experimental design protocol of the two objects exploration and novel object recognition (n=6-8 animals per genotype). (**B**) During training for object recognition, two objects were presented to a mouse during 10 min. WT and *Scrib1$^{crc/+}$* mice spend the same time to explore the objects (no object effect: $F_{1,24}$ < 1, n.s; no genotype effect: $F_{1,24}$ < 1, n.s). (**C**) One hour after, one of the previous objects was changed by a novel object. Recognition memory measured the time spend sniffing the novel object versus the familiar object in the first 5 min of testing. Here two groups of mice presented no difference in recognition memory (Object effect: $F_{1,24}$ = 12.84, ** P < 0.01; no genotype effect: $F_{1,24}$ < 1, n.s; no genotype x object interaction: $F_{1,24}$ < 1, n.s). All data are presented ±SEM. The link hook is used for significant object effect.

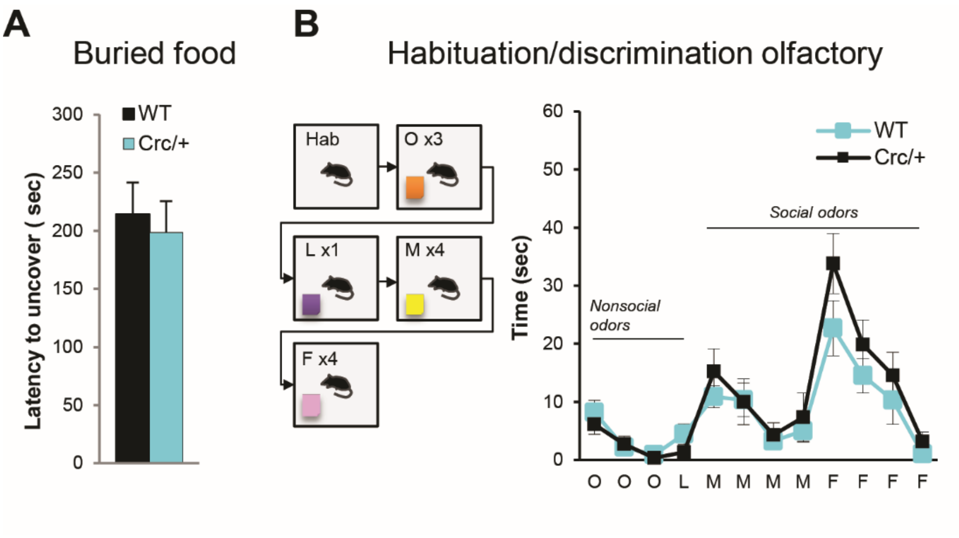

Supplementary Figure S3. The social behavior deficit of the *Scrib1*$^{crc/+}$ mice is not due to abnormal olfactory problems. (**A**) Buried food test. The time required to locate the food buried under the sawdust was similar for both genotypes (WT vs. *Scrib*$^{crc/+}$, *t*-test, t_{26} = 0.40, n.s ; n=13-15 animals per genotype). (**B**) Experimental design protocol of habituation/dishabituation olfactory test. Habituation / dishabituation olfactory test was used to assess sense of smell. Non-social odor was presented following by social odors and mice were assessed for dishabituation on first presentation of novel odor and habituation on the third/four presentation of the odor. Both genotypes displayed similar levels of habituation indicated by a decrease in the time spent sniffing the odorant stimulus following its repeated presentation and comparable levels of dishabituation, indicated by increased time sniffing a novel odorant stimulus (RMANOVA : no genotype effect: $F_{1,187}$ = 1.87, n.s; odor effect: $F_{11,187}$ = 22.54, $p < 0.0001$; no odor x genotype interaction: $F_{11,187}$ = 1.30, n.s; n=9-10 animals per genotype). All data are presented ±SEM.

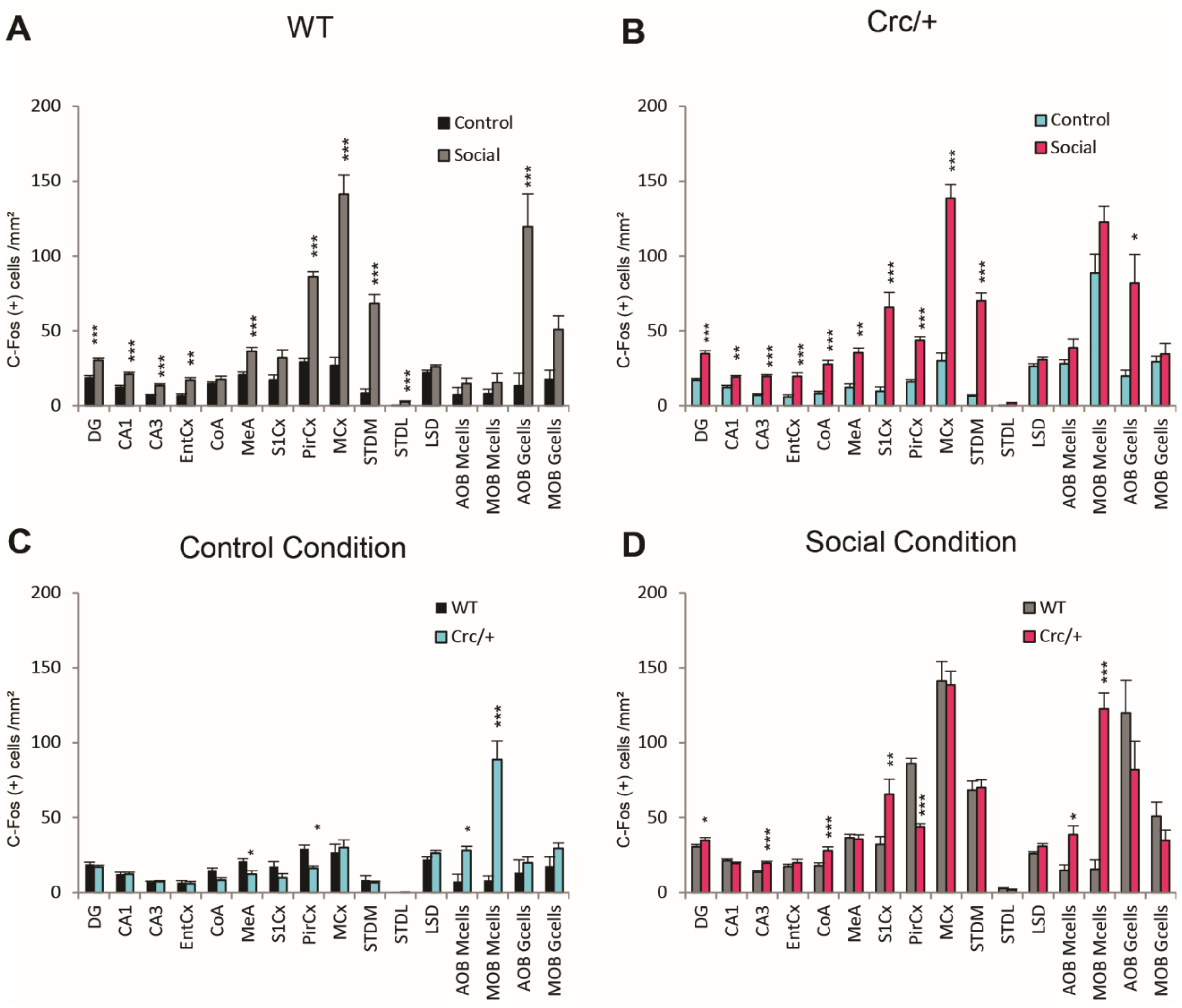

Supplementary Figure S4. Measure of change in c-Fos immunoreactive cells. In response to social test (**A**) in the WT and (**B**) *Scrib1*$^{crc/+}$ mice. (**C-D**) Measure of change in c-Fos immunoractive cells in response to Scrib1 mutation in (**C**) the control condition and (**D**) within the social interest test. Animals per condition (n=3-6); numbers of values per areas: DG (n=25-46), CA1 (n=22-60), CA3 (n=20-57), EntCx (n=13-44), CoA (n=14-28), MeA (n=15-25), S1Cx (n=28-96); PirCx (n=28-96), MCx (n=32-63), STDM (n=35-68), STDL (n=35-64), LSD (n=35-64), AOB Mcells (n=6-13), MOB Mcells (n=15-39), AOB Cells (n=9-13), MOB Cells (n=14-41), One way ANOVA: *$P \leq 0.05$; ** $P \leq 0.01$ and ***$P \leq 0.001$.

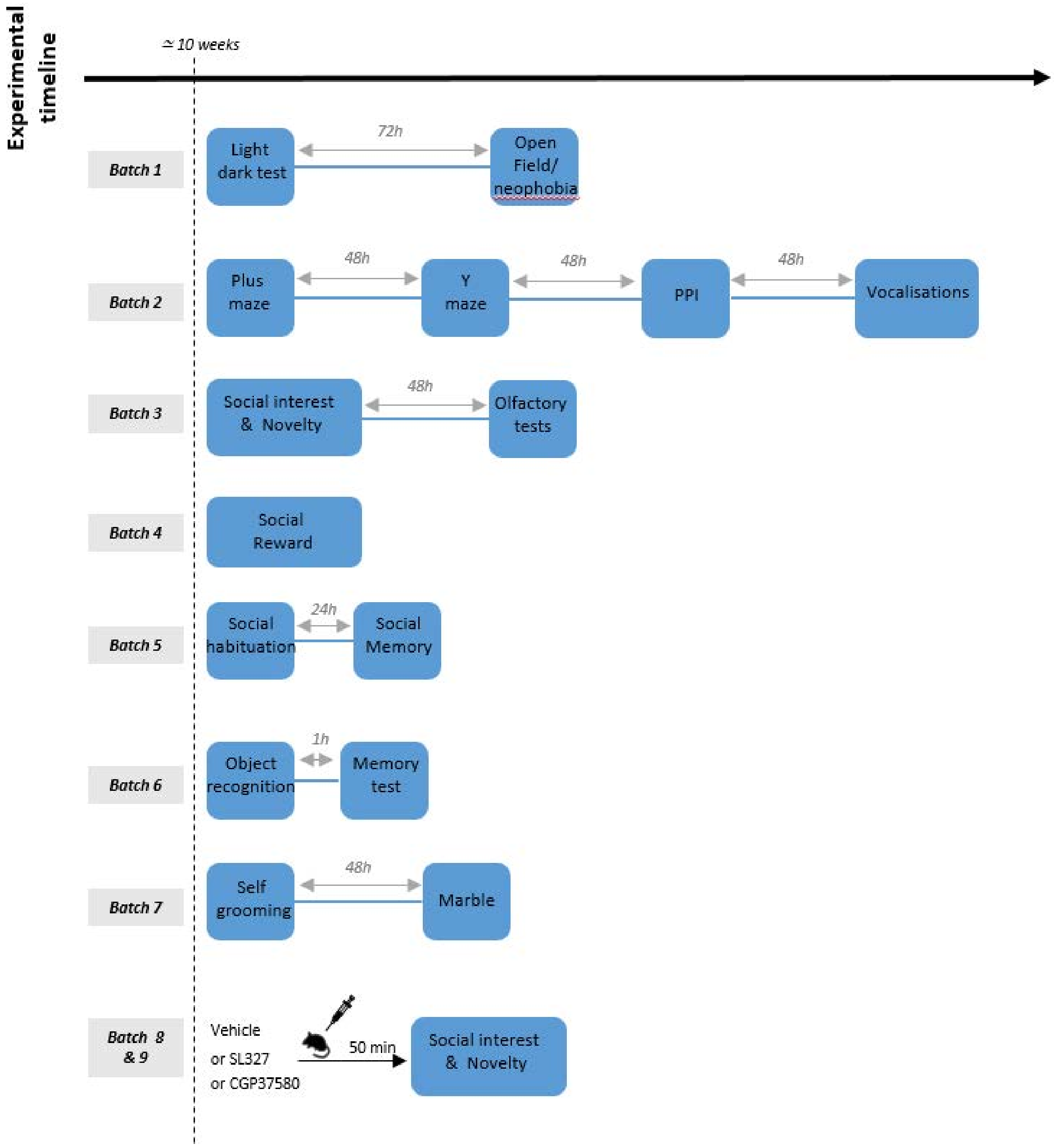

Supplementary Figure S5: Experimental timeline of different behavioural group of mice.

.

Full Blots.

Erk1/2

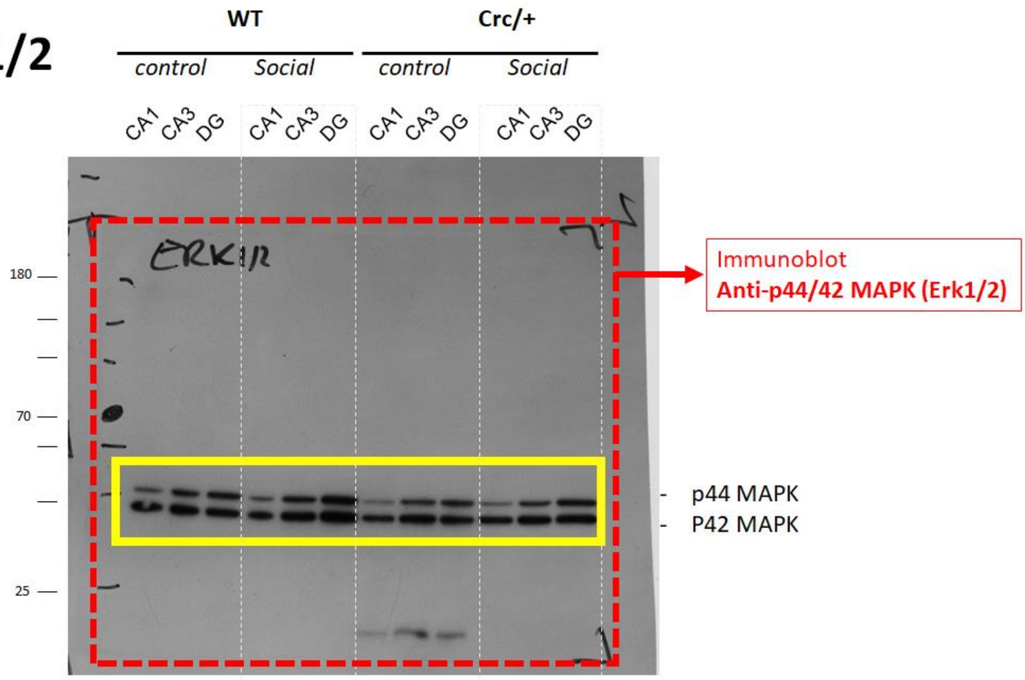

Anti-p44/42 MAPK (Erk1/2)

Phospho-Erk1/2

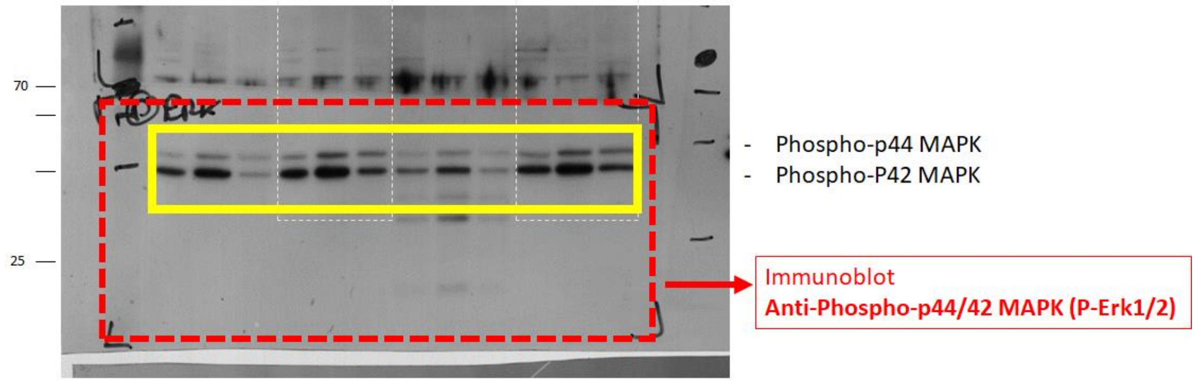

Anti-Phospho-p44/42 MAPK (P-Erk1/2)

Supplementary Figure S6 : Immunoblots of ERK1/2 and P-ERK1/2 from DG, Ca3 AND CA1 of WT and *Scrib1*$^{crc/+}$ mice.
